# Mode-cleaning in antisymmetrically modulated non-Hermitian waveguides

**DOI:** 10.1515/nanoph-2023-0713

**Published:** 2024-01-12

**Authors:** Mohammad Nayeem Akhter, Muriel Botey, Ramon Herrero, Kestutis Staliunas

**Affiliations:** Department de Fisica, Universitat Politecnica Catalunya, Rambla Sant Nebridi 22, 08222, Terrassa, Barcelona, Spain; ICREA, Passeig Lluís Companys 23, 08010, Barcelona, Spain; Faculty of Physics, Laser Research Center, Vilnius University, Sauletekio Ave. 10, 10223, Vilnius, Lithuania

**Keywords:** mode-cleaning, non-Hermitian, waveguides

## Abstract

We demonstrate all-optical spatial mode-cleaning in non-Hermitian waveguides. The effect is accounted by a unidirectional coupling among the modes resulting from a simultaneous modulation of the refractive index and the gain/loss along graded index multimodal waveguides. Depending on the spatial delay between the real and imaginary part of the potential modulation, higher or lower order modes are favored, which in latter case eventually leads to an nearly-monomode propagation. In this way, for any arbitrary initial field distribution an antisymmetric non-Hermitian modulation results in an effective mode-cleaning. The effect is demonstrated analytically, based on coupled mode theory in 1D waveguides, and numerically proven by solving the wave propagation equation with the antisymmetric non-Hermitian potential. The proposal is also generalized to the more involved case of 2D waveguides, leading to a significant reduction of the beam quality factor and improvement of beam spatial quality.

## 1 Introduction

Non-Hermitian systems have boosted attention in photonics, since gain and loss are natural ingredients that can be integrated to design artificial materials holding unconventional properties in the generation, transportation, manipulation and transmission of light [1], [2], [3], [4]. In particular, non-Hermiticity has brought the opportunity to control the modal coupling, for instance to manipulate the flow of light [5], [6], stabilize multimode lasers [7], [8], or for asymmetric two-mode switching in waveguides [9], among others. Precisely in guided wave optics great efforts have been made to achieve tailored gain and loss platforms for efficient realizations, as for instance for non-Hermitian asymmetric mode transfer [10], [11], [12].

In turn, the idea that non-Hermitian spatiotemporal modulation of the background potential can strongly influence the mode dynamics was recently proposed in Ref. [13]. Periodic potentials in space and time in the form *V*(*x*, *t*) ∼ cos(*qx*)[*m*_re_ cos(Ω*t*) + *im*_im_ cos(Ω*t* + *ϕ*)] can unconventionally affect the field dynamics. Depending on the spatial delay between real and imaginary parts of the potential modulation, *ϕ*, either the higher or lower order modes are favored. Indeed in the simplest case *ϕ* = *π*/2, *m*_re_ = *m*_im_ = *m*, the potential reads as $V\left(x,t\right)∼m\text{cos}\left(qx\right)\exp\left(\text{i}\Omega t\right)=m\exp\left(\text{i}\Omega t\right)\left[\exp\left(+\text{i}qx\right)+\exp\left(-\text{i}qx\right)\right]/2$ . Such potential couples the modes with (*k*, *ω*) into (*k* ± *q*, *ω* + Ω). This results in a bidirectional (reciprocal) mode coupling in wavenumber $\left(k\right)$ domain however unidirectional (nonreciprocal) mode coupling in frequency (*ω*) domain. In Ref. [13], this asymmetric property of the non-Hermitian potential has been applied to control the turbulence cascades through the spatial scales. Analogously, the approach allows the control of the modes in parabolic multimode optical fibers [14]. Upon the introduction of such a non-Hermitian modulation, the excitations of Gauss-Laguerre modes with the same helicity *LG*_00_, *LG*_10_, *LG*_20_… can be condensed towards the excitations of lowest order mode, *LG*_00_. Alternatively, depending on the value of *ϕ*, excitations can cascade towards higher order modes. Therefore, the procedure in Ref. [14] works for some specific modes only and does not allow a general and effective mode-cleaning of the propagating beam.

In the present paper, we show that particular non-Hermitian modulation in one- and two-dimensional (2D) waveguides can indeed control all modes, and can result in effective mode-cleaning for any arbitrary initial field distribution. Efforts to tackle issues related to the randomization in optical fibers have been reported involving different techniques [15], [16], [17]. A recently uncovered interesting effect, the so-called beam self-cleaning, was proposed in Ref. [18] and further explored in Refs. [19], [20]. However, this nonlinear mechanism relays on very high intensities, and may not directly lead to a reduction of the beam quality parameter *M* ^2^.

**Figure 1:**
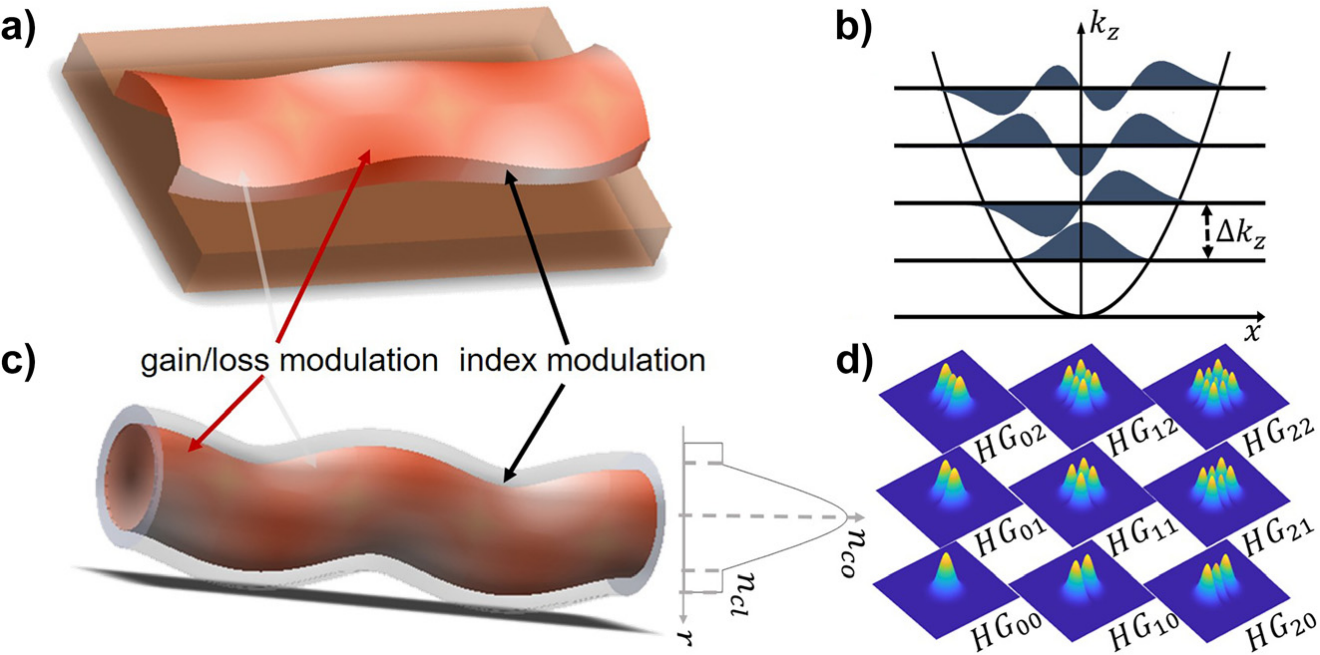
Antisymmetrically modulated non-Hermitian waveguides. (a) Schematic representation of a periodically modulated non-Hermitian 1D waveguide with a parabolic index profile, with a central symmetry axis. The index modulation along the propagation direction is accounted by the fiber snaking, resulting in a transverse antisymmetric index perturbation. The gain/loss also antisymmetric in the transverse direction as indicated by colours. (b) Lowest order Hermite modes of the 1D (unmodulated) parabolic waveguide assuming fundamental mode in transverse direction. (c) Periodically modulated non-Hermitian 2D waveguide, with index and gain/loss modulation along the waveguide. (d) Lowest order Hermite–Gauss modes of the 2D (unmodulated) parabolic waveguide.

We first start analytically exploring the action of a non-Hermitian potential in 1D waveguides with a transverse antisymmetric profile, as illustrated in Figure 1. We generalize the idea to the more involved case of 2D waveguides.

## 2 Model

Light propagation along parabolic refraction index 2D waveguides in paraxial approximation is described by a linear Schrödinger equation:

(1) $$\frac{\partial A}{\partial z}=i\frac{1}{2}\nabla^{2}A-i\frac{\Delta }{r_{c}^{2}}\left(x^{2}+y^{2}\right)A+iV\left(x,y,z\right)A$$

where $A\left(x,y,z\right)$ is the complex field amplitude envelope. The space coordinates are normalized to $k_{0}^{-1}=\lambda /2\pi$ ; being *k*_0_ = *ω*_0_ *n*_co_/*c* the light wavenumber, ∇^2^ = ∂^2^/∂*x* ^2^ + ∂^2^/∂*y* ^2^ is the Laplacian in transverse space, *r*_*c*_ is the core radius, $\Delta =\left(n_{\text{co}}^{2}-n_{\text{cl}}^{2}\right)/\left(2n_{\text{co}}^{2}\right)$ the relative index difference, and *n*_co_ (*n*_cl_) the refractive index of the waveguide core (cladding), and *V*(*x*, *y*, *z*) is the applied non-Hermitian potential. We neglect frequency dispersion effects (as either continuous wave or sufficiently long pulses are considered), and neither include nonlinear effects and Raman scattering.

In the absence of modulation, for $V\left(x,y,z\right)=0$ , light beams propagating in a parabolic waveguide exhibit a periodic self-imaging phenomenon along the fiber, due to the equal spacing of the propagation constant of the modes. The mode spacing (Δ*k*_*z*_) and the self-imaging period (*ζ*) are respectively given by:

(2) $$\Delta k_{z}=\frac{\sqrt{2\Delta }}{r_{c}},\;\;\;\;\;\zeta =\frac{2\pi }{\Delta k_{z}}=\frac{\pi r_{c}}{\sqrt{2\Delta }}$$

## 3 Analytics

In order to analytically asses the interaction among modes for the 1D case, we derive an approximate model, assuming a driven antisymmetric potential with longitudinal frequency *q* close to Δ*k*_*z*_. The linear Schrödinger equation in the presence of the antisymmetric non-Hermitian potential in the 1D waveguide, Eq. (1), simplifies into:

(3) $$\frac{\partial A}{\partial z}=id\nabla^{2}A-icx^{2}A+iV\left(x,z\right)A$$

where $d=1/2,c=\Delta /r_{c}^{2}$ . We consider the following antisymmetric potential:

(4) $$\begin{array}{ll} V\left(x,z\right) & =V\left(z\right)V\left(x\right)\\ & =\left[m_{\text{re}}\cos\left(qz\right)+im_{\text{im}}\cos\left(qz+\phi \right)\right]\left(\frac{x}{x_{0}}e^{-\frac{x^{2}}{x_{0}^{2}}}\right) \end{array}$$

where *m*_re_ and *m*_im_ are the amplitude of the refractive index and gain/loss modulations.

In absence of the *z*-dependent potential $V\left(x,z\right)=0$ , the solutions of Eq. (3) are of the form:

(5) $$H_{n}=\frac{1}{\sqrt{\sqrt{\pi }2^{n}n!w_{0}}}H_{n}\left(x/w_{0}\right)\exp\left(-x^{2}/\left(2w_{0}^{2}\right)\right)$$

where *H*_*n*_(*x*) are the Hermite polynomials, with *n* being the non-negative integer mode index.

We expand the solution of the modulated system, $V\left(x,z\right)\neq 0$ , $A\left(x,z\right)$ in terms of the eigenmodes of the unmodulated waveguide:

(6) $$A\left(x,z\right)=\underset{n}{\sum }a_{n}H_{n}\left(x\right){\text{e}}^{-\text{i}nqz}$$

Then, we obtain:

(7) $$\begin{array}{ll} & \sum_{n}\left(\frac{\partial a_{n}}{\partial z}-inqa_{n}\right)H_{n}{\text{e}}^{-\text{i}nqz}\\ & \;=\sum_{n}\left(id\nabla^{2}-icx^{2}\right)a_{n}H_{n}{\text{e}}^{-\text{i}nqz}\\ & \;+\sum_{n}i\left(m_{+}{\text{e}}^{\text{i}qz}+m_{-}{\text{e}}^{-\text{i}qz}\right)\frac{x}{x_{0}}{\text{e}}^{-\frac{x^{2}}{x_{0}^{2}}}a_{n}H_{n}{\text{e}}^{-\text{i}nqz} \end{array}$$

where *m*_+_ = *m*_re_ + *im*_im_e^i*ϕ*^, *m*_−_ = *m*_re_ + *im*_im_e^*−*i*ϕ*^.

Multiplying by $H_{m}\left(x\right)$ , integrating over the space, applying the orthogonality and normalization conditions of *H*_*n*_, ∫*H*_*n*_ *H*_*m*_d*x* = *δ*_*nm*_, Eq. (7) becomes:

(8) $$\begin{array}{ll} \frac{\partial a_{m}}{\partial z}{\text{e}}^{-\text{i}mqz} & =a_{m}\left(i\beta_{m}+imq\right){\text{e}}^{-\text{i}mqz}\\ & \;+\sum_{n}i\left(m_{+}{\text{e}}^{\text{i}qz}+m_{-}{\text{e}}^{-\text{i}qz}\right)a_{n}C_{nm}{\text{e}}^{-\text{i}nqz} \end{array}$$

here $\beta_{m}=-\frac{\left(m+1\right)\sqrt{2\Delta }}{r_{c}}$ are propagation eigenvectors, and $C_{nm}=\int H_{n}H_{m}\frac{x}{x_{0}}{\text{e}}^{-\frac{x^{2}}{x_{0}^{2}}}dx$ are mode coupling coefficients.

Next, collecting the terms with coefficients e^−i*mqz*^ from both the sides of Eq. (8), we derive the chain of coupled equations with couplings to the nearest neighboring modes. Such truncation is justified by the fact that *C*_*nm*_ = 0 for *m* = *n* and *m* = *n* ± 2, while *C*_*m*±3,*m*_ ≪ *C*_*m*±1,*m*_ due to the symmetry of Hermite polynomials.

(9) $$\begin{array}{ll} \frac{\partial a_{m}}{\partial z} & =a_{m}\left(i\beta_{m}+imq\right)+im_{+}C_{m+1,m}a_{m+1}\\ & \;+im_{-}C_{m-1,m}a_{m-1} \end{array}$$

This expansion results extremely useful when *β*_*m*_ ≃ −*mq*, i. e. close to the resonances, and we write Δ*q*_*m*_ = *β*_*m*_ + *mq*.

The general coupling matrix for *n* modes, as derived from Eq. (9) is expressed as:

(10) $$\left[\begin{array}{ccccc} i\Delta q_{1} & im_{+}C_{21} & 0 & \ldots & 0\\ im_{-}C_{12} & i\Delta q_{2} & im_{+}C_{32} & \ldots & 0\\ 0 & im_{-}C_{23} & \ddots & \vdots & \vdots \\ \vdots & \vdots & \vdots & \ddots & im_{+}C_{n,n-1}\\ 0 & 0 & \ldots & im_{-}C_{n-1,n} & i\Delta q_{n} \end{array}\right]$$

**Figure 2:**
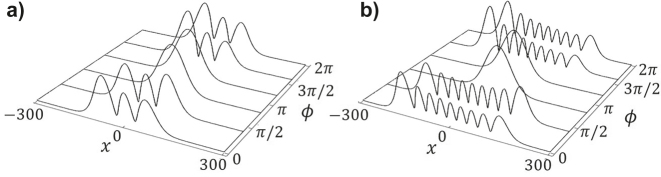
Transverse profile of the eigenmode with largest eigenvalue assuming: (a) 3 modes system, (b) 9 modes system. Parameters used: *m*_re_ = *m*_im_ = 1.5 × 10^−4^, *q* = 0.9Δ*k*_*z*_ (below resonance).

In order to determine the eigenvalues of this coupling, we consider a reduced number of modes, up to *n* = 9. The real part of eigenvalues determines the growth rates of the corresponding eigenvectors, defining the mode composition in the locked-mode state. After a sufficiently long propagation along the fiber, we expect the field profile to be the eigenvector with largest positive real part of the eigenvalue. As an example, Figure 2a and b presents such eigenvector as a function of the spatial delay between the real and imaginary modulations, for a truncation of 3 and 9 modes, respectively. Importantly, in both cases, and even for the 3-modes approach, we observe a wide range, for *ϕ* ≈ *π* with a bell-shaped profile. Therefore, we expect that, for such parameters, a beam propagating along the fiber evolves towards this shape, irrespectively of the initial input beam. We note that for *ϕ* = *π*/2 and 3*π*/2 the real parts of the eigenvalues are 0, therefore no effect is expected. Finally, we note that for −*π*/2 < *ϕ < π*/2 the eigenmode profile indicates the participation of higher order modes, see Figure 2a and b. Therefore, irrespectively of the initial shape of the propagating beam, high order modes are expected to be predominant in propagation along the waveguide.

## 4 Results

### 4.1 1D non-Hermitian waveguide

For the simplest case, the 1D system is described by Eq. (3), and the eigenmodes of the modulated system may be expanded in terms of the Hermite polynomials, Eq. (6). We introduce the potential of Eq. (4) with an antisymmetric shape in *x,* the transverse direction. In order to characterize the mode coupling we calculate the relative intensity as the overlap integral (OI) of the relative modes to the total field, *A*(*x*)*,* in linear scale given by:

(11) $${\text{OI}}_{n}=\frac{{\left|\int A\times H_{n}dx\right|}^{2}}{\int {\left|A\right|}^{2}dx\times \int {\left|H_{n}\right|}^{2}dx}$$

**Figure 3:**
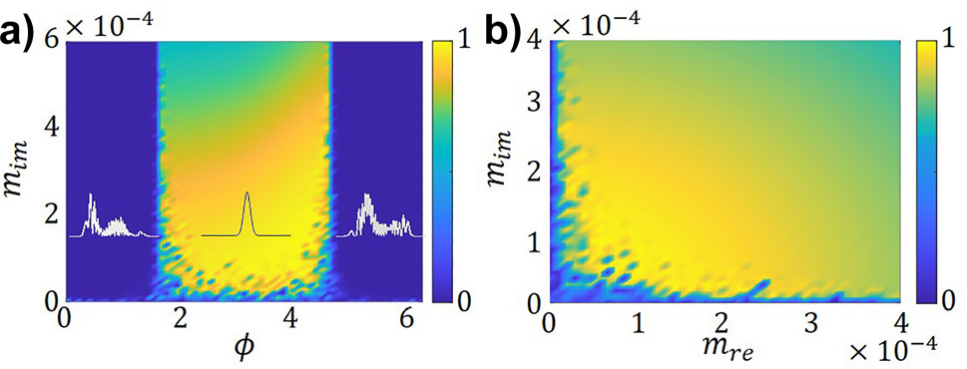
Map of the participations the lowest mode, *H*_0_, in the total field, after propagation along 3·10^3^ self-imaging periods: (a) in the parameter space (*ϕ*, *m*_im_) for a fixed *m*_re_ = 1.5 × 10^−4^, (b) in the parameter space (*m*_re_, *m*_im_) for a fixed *ϕ* = *π*; for both maps upon incidence of a noisy multimodal beam. The insets in figure a) present the final field profile for three situations, *ϕ* = ±*π*/4, *π* where *m*_im_ = 1.5 × 10^−4^ and *q* = 0.9Δ*k*_*z*_.

To assess the mode-cleaning effect, we first numerically explore the parameter space of (*ϕ*, *m*_im_) for a fixed value of *m*_re_. We propagate along the fiber an initially random beam, and map the participation of the lower order mode, *H*_0_, after a sufficiently long propagation distance, namely 3·10^3^ self-imaging periods, see Figure 3a. Indeed, we observe a region, near *ϕ* = *π*, and for a small, *m*_Im_ < 0.0002, where the *H*_0_ mode participation is maximized. In the following calculations, we will consider this range of parameters. Note that, in systems near resonance the effect is shifted by *π*/2 [14]. Notably, we observe condensation of energy in the lowest mode, mode-cleaning, for moderate amplitudes of the modulations of the refractive index and gain/loss. Besides such feasible conditions, the effect seems robust over a wide range of values, see Figure 3b.

**Figure 4:**
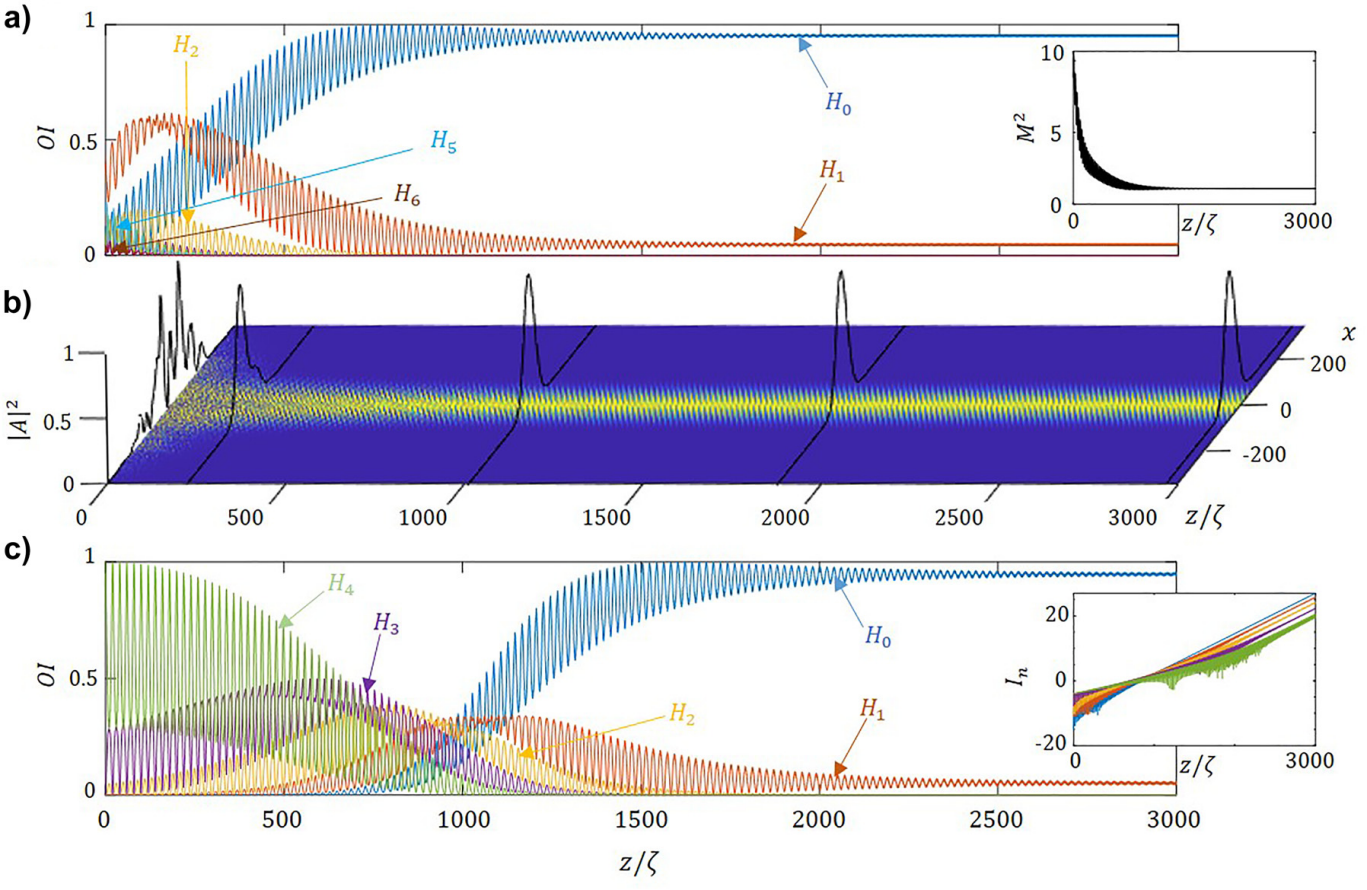
Mode-cleaning in 1D waveguide. (a) Relative mode intensities, OI, for an incident noisy beam as a function of the propagation distance, normalized to the self-imaging period. The inset shows the evolution in space of the beam quality factor. (b) Evolution in propagation of the corresponding with profiles depicts at particular distances, namely: *z* = *ζ*, 230*ζ* *,* 1020*ζ*, 1920*ζ*, and 2990*ζ*. (c) Relative mode intensities, OI, for an incident monomode *H*_4_ as a function of the propagation distance, normalized to the self-imaging period. The inset shows the modal growth of the lower five modes. Parameters used in the numerical integration: *m*_re_ = *m*_im_ = 1.5 × 10^−4^, *ϕ* = *π*, *q* = 0.9Δ*k*_*z*_.

Figure 4a shows the participation of the modes in propagation along the fiber for a multimode input for parameter of the modulated non-Hermitian fiber within the mode-cleaning regime. Note that the lowest order mode (*H*_0_) increases asymptotically approaching to unity as the beam propagates along the fiber, while the participation of higher order modes decreases. The inset depicts the evolution of the beam quality factor, *M* ^2^, which decreases towards 1. In turn, Figure 4b shows the evolution of intensity in propagation. The highly multimodal input distribution of the beam gradually evolves towards a bell-shaped transverse profile. Note that for −*π*/2 ≤ *ϕ* ≤ *π*/2 the effect is reversed and the relative participation of the lowest order modes decreases, as the higher order modes become predominant.

Finally, we show the effect holds even when the initial beam does not contain the lowest order mode. As an example, we provide a particular situation: we start with the *H*_4_ mode at the input of the fiber, and we observe that as light propagates along the fiber, the participation of other modes appears, as energy cascades towards lower order modes and finally condensates in the lowest one, *H*_0_. Figure 4c depicts the relative intensity of the five lower order modes, we observe the participation of *H*_0_ mode almost reaches 1. The system may be characterized by a second figure of merit; the absolute modal intensity of in logarithmic scale as:

(12) $$I_{n}=\log{\left|\frac{\left|\int A\times H_{n}^{*}\text{d}x\right|}{\int {\left|H_{n}\right|}^{2}\text{d}x}\right|}^{2}$$

which determines the growth rate of every particular mode, see the inset in Figure 4c. Note, the absolute mode intensity of the *H*_0_ mode grows faster than the other after 10^3^ self-imaging periods. Eventually, all growth rates become equal, upon reaching the eigenstate of the system.

### 4.2 2D non-Hermitian waveguide

Next, we consider the more involved case of 2D waveguide schematically presented in in Figure 1c. In this case the eigenmodes of the 2D fiber may be expanded in terms of the by Hermite–Gauss modes, *HG*_*mn*_, as shown in Figure 1d. The character of the modulation in the *z* direction is assumed to be the same as in the 1D case, but we have several possibilities to modulate in transverse space. In the simplest case, we assume a potential of the form:

(13) $$V\left(x,y,z\right)=V\left(z\right)V\left(x,y\right)=V\left(z\right)\left(\frac{x}{r_{0}}e^{-\frac{\left(x^{2}+y^{2}\right)}{r_{0}^{2}}}\right)$$

**Figure 5:**
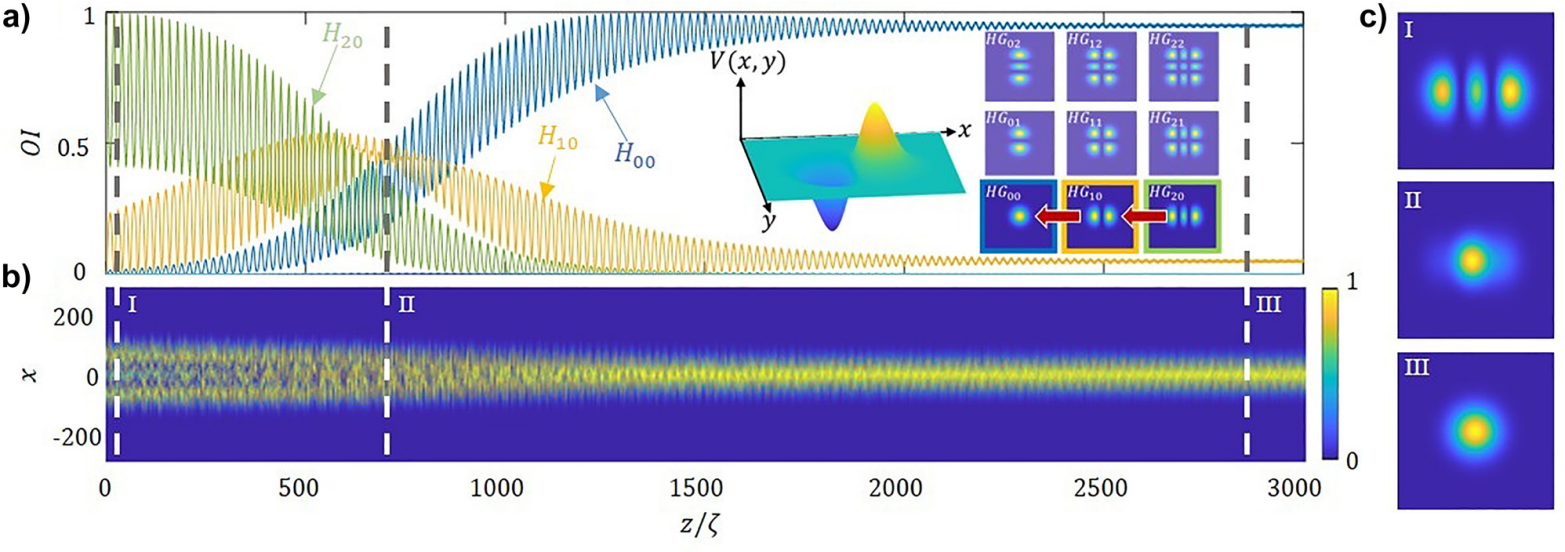
Evolution of mode participations and intensity profile. (a) Relative mode intensities, OI, for an incident monomode *HG*_20_ beam, as a function of the propagation distance, normalized to the self-imaging period. The first inset shows the transverse profile of the applied non-Hermitian potential and the second inset shows the direction of the coupling. (b) Distribution of the propagated intensity along the fiber, the right-hand panels provide the transverses profiles at articular distances, namely: (I) *z* = 5*ζ*, (II) *z* = 670*ζ* and (III) *z* = 2810*ζ*. The parameters used are the same as in Figure 4.

Being the *V*(*z*) the non-Hermitian longitudinal profile of Eq. (4) and *V*(*x*,*y*) a Gaussian transverse profile, of this potential is depicted in the first inset of Figure 5a. We numerically simulate the propagation an incident monomode *HG*_20_ beam, and we observe the unidirectional coupling towards the lower modes along the fiber. As expected, the energy cascades from mode *HG*_20_ to mode *HG*_10_ and *HG*_00_, as shown in the right inset of Figure 5a. Figure 5b provides the numerically propagated 2D evolution, stressing the spatial 2D distributions of the intensity profile at different propagation lengths.

**Figure 6:**
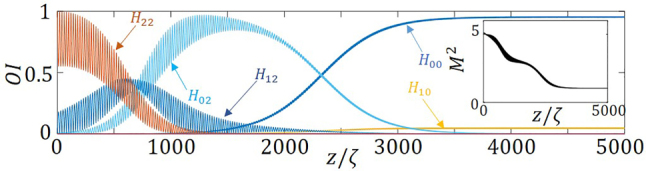
Relative mode intensities, OI, for an incident monomode *HG*_22_, as a function of the propagation distance, normalized to the self-imaging period. The inset shows the evolution in space of the beam quality factor. The parameters used are same as in Figure 4.

Analogously, injecting a monomode *HG*_22_ beam, also leads to a mode-cleaned beam. Figure 6 shows how energy cascades from mode *HG*_22_ to *HG*_12_, to *HG*_02_, and after a sufficient propagation distance *HG*_00_ mode starts growing to become dominant. Note that an unexpected *HG*_02_ → *HG*_00_ transition occurs due to the potential modulation in the *y* direction, through the Gaussian part of the potential, *V*(*x*, *y*), in Eq. (13). To characterize it we also calculate the beam quality factor, *M* ^2^, which gradually decreases to unity, as can be seen in the inset of Figure 6.

The *x*-multiplying factor of the potential couples every neighboring mode in the *x* direction with frequency difference *q* (near Δ*k*_*z*_), while in the *y* direction every second mode with frequency difference 2*q* is coupled because of the Gaussian potential as discussed in Ref. [14].

**Figure 7:**
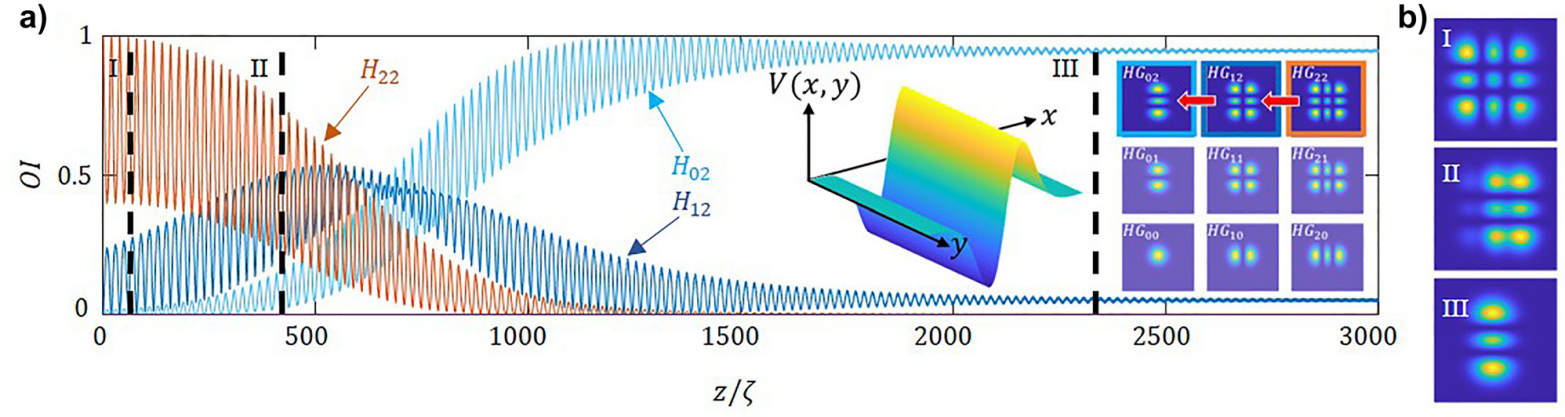
Evolution of mode participations. (a) Relative mode intensities, OI, for an incident monomode *HG*_22_, as a function of the propagation distance, normalized to the self-imaging period. The first inset shows the transverse profile of the applied non-Hermitian potential and the second inset shows the direction of the coupling. (b) The panels on the right provide the 2D transverse distributions of the corresponding at particular distances, namely: (I) *z* = 25*ζ*, (II) *z* = 470*ζ*, and (III) *z* = 2370*ζ*. The parameters used are same as in Figure 4.

To better understand this *HG*_02_ → *HG*_00_ transition we consider a potential uniform in the *y* direction as:

(14) $$V\left(x,z\right)=V\left(z\right)\left(\frac{x}{r_{0}}e^{-\frac{x^{2}}{r_{0}^{2}}}\right)$$

which is antisymmetric in *x* but uniform in *y*, as shown in the first inset of Figure 7a. Figure 7 summarizes the scenario of the evolution of mode *HG*_22_ under the potential (14). The relative mode intensities of the *HG*_22_, *HG*_12_ and *HG*_02_ modes decrease along the fiber, while we do not observe the participation of the mode *HG*_00_, mode as in the previous case. Only a unidirectional cascade coupling from mode *HG*_22_ to *HG*_12_, and to *HG*_02_ is observed, and it is this last mode, which relative intensity almost reaches 1. No transition from *HG*_02_ to *HG*_00_ is induced in this case, as shown in Figure 7. This is also evident from the 2D transverse profiles of the propagated beam at some particular propagation distances, provided in Figure 7b.

In turn, a potential being antisymmetric in the *y* direction as

(15) $$V\left(y,z\right)=V\left(z\right)\left(\frac{y}{r_{0}}e^{-\frac{y^{2}}{r_{0}^{2}}}\right)$$

yields the cascading *HG*_22_ → *HG*_21_ → *HG*_20_ as an analogous effect of the previously described antisymmetric case in *x* transverse direction.

**Figure 8:**
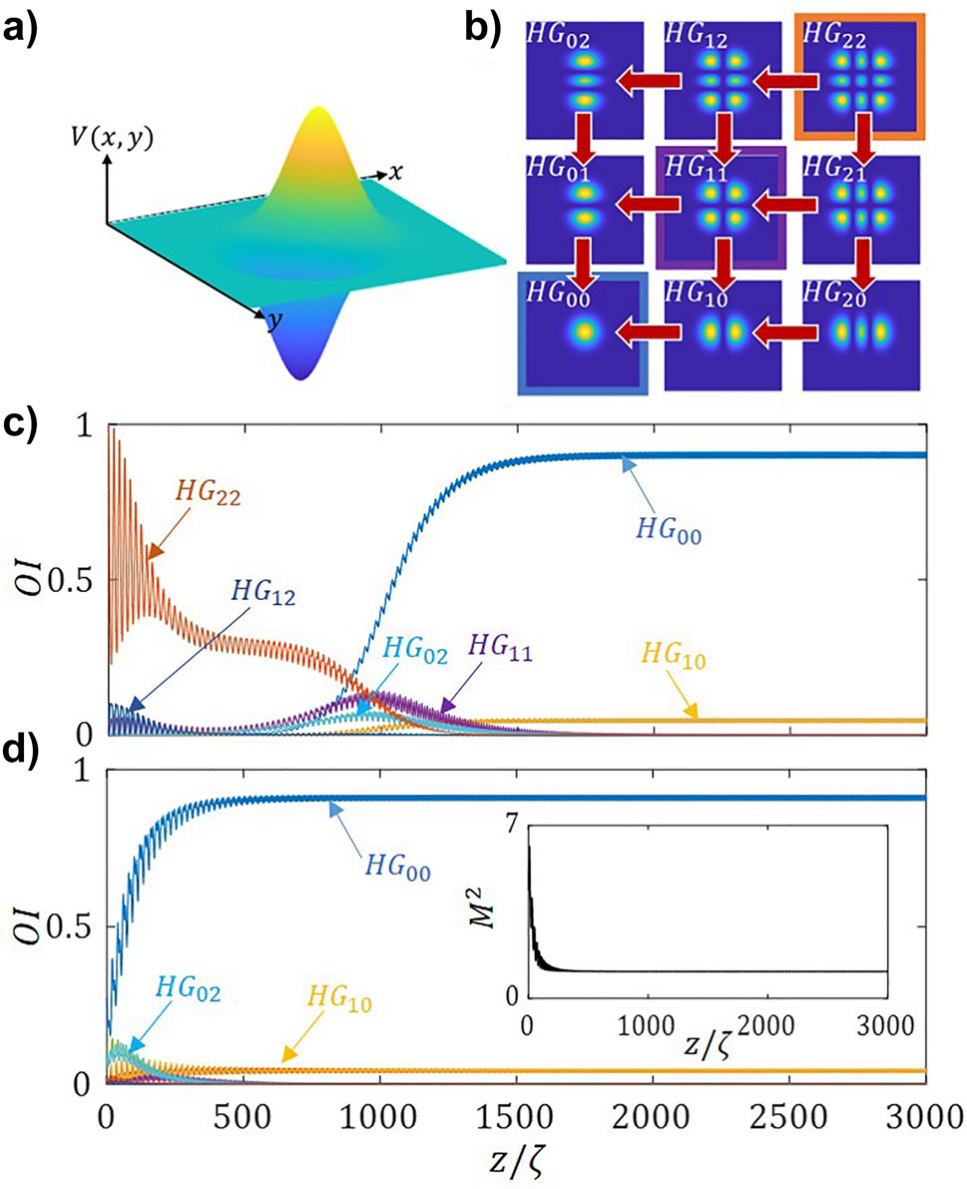
2D mode-cleaning. (a) Transverse profile of the applied non-Hermitian potential in Eq. (16). (b) Visualization of the mode unidirectional coupling both directions. Relative mode intensities, OI, as a function of the propagation distance, normalized to the self-imaging period. For: (c) an incident monomode *HG*_*22*_ beam, and (d) upon a noisy random input. The parameters used are same as in Figure 4. Note in (c) (*HG*_12_ and *HG*_21_), (*HG*_02_ and *HG*_20_) and (*HG*_01_ and *HG*_10_) are superposed.

Next, we apply a diagonal potential of the form:

(16) $$V\left(x,y,z\right)=V\left(z\right)\left(\frac{x+y}{r_{0}}e^{-\frac{\left(x^{2}+y^{2}\right)}{r_{0}^{2}}}\right)$$

with a transverse profile as shown in Figure 8a. Such a potential is expected to couple mode *HG*_22_ simultaneously to modes *HG*_12_ and *HG*_21_, as shown by the red arrows in Figure 8b by the red arrows. Indeed, Figure 8c shows the relative mode intensities, OI, for an incident monomode *HG*_22_ beam, as a function of the propagation distance. We observe such coupling in both the *x* and y directions effectively leads to a predominance of the *HG*_00_ mode, as the other relative mode intensities rapidly decrease. Note that due to the *x*–*y* symmetry, the plots corresponding to modes (*HG*_12_ and *HG*_21_), (*HG*_02_ and *HG*_20_) and (*HG*_01_ and *HG*_10_) share the same evolution, and therefore appear superposed in Figure 8c.

Upon the incidence of a noisy beam, the same potential (16) effectively converges to the eigenmode, thus significant mode-cleaning is observed see the inset in Figure 8d, as the beam quality factor, *M* ^2^, rapidly decreases to 1.

In turn, a potential such as:

(17) $$V\left(x,y,z\right)=V\left(z\right)\left(\frac{x\pm iy}{r_{0}}e^{-\frac{\left(x^{2}+y^{2}\right)}{r_{0}^{2}}}\right)$$

induces a cascading energy transfer from any mode *HG*_*m,n*_ to mode *HG*_*m*−1,*n*+1_. The real part of the potential induces a coupling towards modes with smaller *m* index, while the imaginary part yields to higher *n* index.

Throughout the paper we assume a quasi-resonant non-Hermitian potential, being *q* just below Δ*k*_*z*_. We finally investigate the effect of shifting *q* above resonance and observe a sharp reversed effect; in this situation mode-cleaning occurring for *ϕ* ≈ 0.

In turn, we performed an analysis of the robustness of the mechanism. We observe that the scheme is effective and mode-cleaning persists for small perturbations from the parabolic index profile, while in this case the modes are no more equidistant.

The actual fabrication of the proposed non-Hermitian 1D waveguides could be achieved in 1D using current lithographic techniques [12], [21], [22], [23]. The required index and gain/loss modulation in 2D waveguides may still be feasible for instance by doping the fiber core [24], [25] and introducing distributed absorption [26], scattering [27], or transmission losses [28].

## 5 Conclusions

In this work, we demonstrate a mode-cleaning in 1D and 2D waveguides with periodic non-Hermitian perturbation of the potential. The effect is predicted analytically in 1D, from the eigenvectors of the mode-coupling matrix using a truncated mode expansion. Irrespectively of the incident beam, the resulting beam upon propagation in the non-Hermitian antisymmetric waveguide will resemble the locked-mode state. We map the parameter space by numerically integrating the nonlinear Schrödinger equation, finding a good agreement with analytics. For particular characterizing parameters of the non-Hermitian potential, the relative intensity of the lowest Hermite mode being close to 1. We also extend the proposal to 2D waveguides. Indeed, we numerically demonstrate an efficient mode-cleaning, for different geometries of the antisymmetric transverse potential leading to a reduction of the beam quality factor close to 1. We observe that the longitudinal phase delay and the detuning from resonance are two important parameters of the non-Hermitian potential. They both can induce a switch in the behavior, leading to either the predominance of higher or lower order modes in the mode composition in eigenmode. The last situation corresponding to the demonstrated mode-cleaning which may be efficiently realized with the actual nanofabrication techniques, and attained within a length of only few hundreds of self-imaging periods.
